# An experimental investigation of a novel iron chelating protoporphyrin IX prodrug for the enhancement of photodynamic therapy

**DOI:** 10.1002/lsm.22809

**Published:** 2018-03-31

**Authors:** Lizette Anayo, Anette Magnussen, Alexis Perry, Mark Wood, Alison Curnow

**Affiliations:** ^1^ European Centre for Environment and Human Health University of Exeter Medical School University of Exeter Environment and Sustainability Institute Penryn Campus Cornwall TR10 9FE UK; ^2^ Biosciences, College of Life and Environmental Sciences University of Exeter Geoffrey Pope Building, Stocker Road Exeter Devon EX4 4QD UK

**Keywords:** aminolaevulinic acid (ALA), AP2‐18, CP94, dermatology, iron chelation, methyl‐aminolevulinate (MAL), photodynamic therapy (PDT), protoporphyrin IX (PpIX), pyridinone, skin

## Abstract

**Objectives:**

Non‐melanoma skin cancers are the most frequently occurring type of cancer worldwide. They can be effectively treated using topical dermatological photodynamic therapy (PDT) employing protoporphyrin IX (PpIX) as the active photosensitising agent as long as the disease remains superficial. Novel iron chelating agents are being investigated to enhance the effectiveness and extend the applications of this treatment modality, as limiting free iron increases the accumulation of PpIX available for light activation and thus cell kill.

**Methods:**

Human lung fibroblasts (MRC‐5) and epithelial squamous carcinoma (A431) cells were treated with PpIX precursors (aminolaevulinic acid [ALA] or methyl‐aminolevulinate [MAL]) with or without the separate hydroxypyridinone iron chelating agent (CP94) or alternatively, the new combined iron chelator and PpIX producing agent, AP2‐18. PpIX fluorescence was monitored hourly for 6 hours prior to irradiation. PDT effectiveness was then assessed the following day using the lactate dehydrogenase and neutral red assays.

**Results:**

Generally, iron chelation achieved *via* CP94 or AP2‐18 administration significantly increased PpIX fluorescence. ALA was more effective as a PpIX‐prodrug than MAL in A431 cells, corresponding with the lower PpIX accumulation observed with the latter congener in this cell type. Addition of either iron chelating agent consistently increased PpIX accumulation but did not always convey an extra beneficial effect on PpIX‐PDT cell kill when using the already highly effective higher dose of ALA. However, these adjuvants were highly beneficial in the skin cancer cells when compared with MAL administration alone. AP2‐18 was also at least as effective as CP94 + ALA/MAL co‐administration throughout and significantly better than CP94 supplementation at increasing PpIX fluorescence in MRC5 cells as well as at lower doses where PpIX accumulation was observed to be more limited.

**Conclusions:**

PpIX fluorescence levels, as well as PDT cell kill effects on irradiation can be significantly increased by pyridinone iron chelation, either *via* the addition of CP94 to the administration of a PpIX precursor or alternatively *via* the newly synthesized combined PpIX prodrug and siderophore, AP2‐18. The effect of the latter compound appears to be at least equivalent to, if not better than, the separate administration of its constituent parts, particularly when employing MAL to destroy skin cancer cells. AP2‐18 therefore warrants further detailed analysis, as it may have the potential to improve dermatological PDT outcomes in applications currently requiring enhancement. Lasers Surg. Med. 50:552–565, 2018. © 2018 The Authors. *Lasers in Surgery and Medicine* Published by Wiley Periodicals, Inc.

## INTRODUCTION

Non‐melanoma skin cancers (NMSC) are one of the most common types of cancer Worldwide 1, 2. They are predominantly located on the face and neck, and can lead to local skin damage or disfigurement 3 if left untreated. Although they are not associated with high mortality rates, they occur with high frequency 3 and their occurrence is increasing due to a rise in ultraviolet exposure, a causation factor 1, 3, 4. They are mainly diagnosed in older Caucasians 3, 5 but all age groups are at risk 4. NMSC can be divided into basal cell carcinoma (BCC) and squamous cell carcinoma (SCC) 3, 5. BCC is the most common NMSC and can either be classified as superficial (sBCC) or nodular (nBCC) 3. SCC formation is thought to progress from pre‐cancers like actinic keratosis and Bowen's disease in a small number of cases 3, 6.

Traditionally, NMSCs have been surgically excised, treated with topical 5‐fluorouracil application or frozen with cryotherapy 6, 7, 8, 9. However, these conventional therapies are not always associated with excellent cosmesis 6, 7, 8, 9 and their appropriateness can be limited, depending on the location, size, and number of NMSC lesions to be treated 9, 10, 11. Alternative treatment options have therefore been sought. Photodynamic therapy (PDT) is a relatively new, minimally invasive, light‐induced drug treatment of certain forms of NMSC 5, 6, 7, 8, 9, 10, 11. Dermatological PDT can be administered by nurses and is safe, with few side effects beyond treatment effects 7, 8, 10, 11, 12. Several cancers, including NMSCs and pre‐cancers can be treated using PDT 5, 6, 7, 8, 9, 10, 11, 12, 13, 14. It can also be used to treat some non‐malignant skin diseases such as psoriasis and acne 15. PDT has several advantages over some forms of traditional management, including the possibility of treating a whole area of field change at once, the occurrence of good healing on the lower leg, repeated application to the same area of the skin without the development of resistance, excellent cosmetic results in highly visible sites without the need for advanced surgical techniques and its compatibility as an adjuvant with other treatment approaches 5, 6, 7, 8, 9, 10, 11, 12, 13, 14, 15.

PDT utilizes a light‐absorbing molecule (photosensitizer), activating light of a specific wavelength and molecular oxygen 16, 17, 18. After absorbing energy from the light, the photosensitizer moves into higher energetic states and transfers this additional energy to molecular oxygen directly or indirectly *via* type II or type I photochemical reactions, respectively 16, 17, 18, 19. These reactions form reactive oxygen species (ROS), which then damage cellular components like proteins, lipids, and DNA or indeed the photosensitizer itself, inducing photobleaching 18, 19, 20, 21. The cellular cascades of ROS thus generated, overwhelm the cell's inherent antioxidant defense and ultimately lead to cell death *via* apoptosis and necrosis, or alternatively, a destructive form of autophagy 18, 19, 20, 21, 22, 23, 24, 25.

The photosensitizer most commonly used in dermatological PDT is protoporphyrin IX (PpIX) 10, 11, 13. PpIX (a large, water‐insoluble molecule) can be excited by light of wavelength 635 nm 26. This light penetrates deeper into the tissue than shorter activating wavelengths 27. Skin lesions are treated with a topical cream containing a small, soluble precursor to PpIX (e.g., 5‐aminolaevulinic acid [ALA] or the methyl‐ester of ALA, methyl‐aminolevulinate; MAL)) 10, 11. This is absorbed by cells and enzymatically converted into light sensitive PpIX over a few hours (typically three in clinical application) by the haem biosynthesis pathway naturally present in all nucleated cells 10, 26, 27. This exogenous administration of copious amounts of PpIX precursor bypasses the primary rate limiting step of this pathway (the synthesis of ALA from glycine and succinyl‐CoA by ALA synthase) 26, 27, 28. This forces the rest of the pathway to operate at maximal capacity until PpIX (the immediate precursor to haem) is formed. This naturally light sensitive compound starts to accumulate over time as the final step in the pathway (the insertion of Fe^2+^ into PpIX by ferrochelatase to form haem) is relatively slow to occur and is thus the secondary rate limiting step of this pathway 26, 27, 28.

ALA‐PDT was first introduced experimentally by Malik and Lugaci in 1987 29, with the first clinical treatments reported by Kennedy et al. in 1990 17. It is particularly effective in cancer cells as PpIX accumulation is both lower and slower in normal cells, resulting in less damage to the healthy cells in close proximity to the diseased cells in the treatment area 26. This occurs as haem biosynthesis is elevated and less well controlled in neoplastic cells and tumor cells also have an altered iron metabolism and dysregulated porphyrin biosynthesis enzymes, which makes them more prone to accumulate PpIX more rapidly 26, 30, 31. The disrupted tumor surface is also more permeable than healthy skin, hence facilitating PpIX precursor penetration to where its treatment action is needed most 26, 31.

Although effective treatment outcomes associated with excellent cosmesis have been demonstrated in licensed dermatological lesions (actinic keratosis, Bowen's disease, and BCC) when the disease remains superficial 10, 32, efforts continue to both increase the efficacy and extend the applications of dermatological PDT particularly in order to treat thicker or acrally located conditions 33. It is already known that poor penetration into the deeper skin layers can be improved clinically by employing more lipophilic ALA derivatives (e.g., MAL; Metvix, Galderma, UK) 34, 35, 36, 37, 38, 39, 40 or nanoemulsion formulations (e.g., ALA; Ameluz, Spirit Healthcare, UK) 32 and by performing skin pre‐treatments like the removal of the outer skin layer by tape stripping or scraping 41, 42, 43. In fact many adaptations to the standard treatment protocol have been considered to improve efficacy including skin pre‐treatment with the malignant cell differentiation potentiator dimethyl sulfoxide 44, light dose fractionation 45, 46, low fluence rate light administration 47 as well as combinations with other techniques (e.g., low dose Photofrin 48, hyperthermia 49, 50, iontophoresis 51, and bioreductive drugs 52). Concurrent administration of an iron chelator during PpIX‐PDT has also been demonstrated to increase cellular accumulation of PpIX 20, 53, 54. This approach works because once the PpIX has bound Fe^2+^ to form haem, it is no longer active for PDT and so by adding iron chelators to trap iron ions in combination with PpIX precursors, this competition can be undermined elevating PpIX accumulation and thus increasing cell kill on subsequent irradiation 55, 56. Iron chelation has now been reported as a method of enhancing PpIX‐induced PDT using the iron chelating agents, ethylenediamine tetraacetic acid (EDTA) 41, 42, 43, 57, desferrioxamine (DFO; Desferal, Novartis) 57, 58, 59, 60, 61 and the novel hydroxypyridinone iron chelator CP94 62, 63, 64, 65, 66. This particular method of enhancement is attractive because it is a simple pharmacological modification, which requires no alteration to the licensed treatment protocol other than the addition of the iron chelator to the PpIX prodrug cream 12, 67.

CP94 (1,2‐diethyl‐3‐hydroxypyridin‐4‐one hydrochloride) is a member of the hydroxypyridinone family of oral iron chelators originally developed to supersede DFO (which is a natural siderophore (iron carrier) that has to be administered intravenously clinically *via* long infusion) in the treatment of iron overload. It is closely related to the clinically established oral iron chelating agent Deferiprone (1,2‐dimethyl‐3‐hydroxypyridin‐4‐one hydrochloride (CP20); Ferriprox, Swedish Orphan Biovitrum Ltd, Sweden) but CP94 was found to be superior to CP20 for this particular application (PpIX‐PDT enhancement) in previous animal studies 65. CP94 is particularly effective at chelating intracellular iron and has a lower molecular weight and higher lipophilicity than either DFO or EDTA 68 and is well suited to augmenting dermatological PDT as it can be applied topically 69. CP94 has already been demonstrated to enhance ALA‐induced PpIX fluorescence 62 and to also produce greater tumor necrosis within animal models 65, 66. CP94 has been investigated in a healthy skin explant model producing increased PpIX accumulation when employing either ALA or MAL as the PpIX precursor 63. When the level of PpIX accumulation produced by DFO and CP94 were compared directly *in vitro* 53, these iron chelators in combination with ALA or MAL were shown to significantly increase the amount of PpIX accumulating within fetal lung fibroblasts (MRC5) and the more difficult to culture squamous epidermal carcinoma cells (A431), whilst minimal enhancement was observed in the normal skin fibroblasts (84BR), and keratinocytes (NHEK) investigated. Where enhancement was observed, CP94 was consistently demonstrated to be significantly superior to DFO in the production of elevated PpIX levels 53 and it has already been established that DFO is superior to EDTA in its ability to enhance PpIX‐induced PDT 57.

In clinical studies of iron chelator enhancement of PpIX‐induced PDT, Fijan et al. 59 demonstrated the feasibility of combining the iron chelator DFO with ALA‐PDT to treat 34 superficial and 22 nodular basal cell carcinomas. Liu et al. 42 and Choudry et al. 58 have also investigated EDTA and DFO respectively in humans, utilizing matched skin lesion controls. Lui et al. 42 found a promising and significant (*P* < 0.01) reduction in tumor depth in lesions treated with EDTA in combination with ALA‐PDT. Choudry et al. 58 however, could not detect any significant differences in surface fluorescence between lesions co‐incubated with ALA +/− DFO. Two clinical pilot studies of CP94 in combination with ALA or MAL‐induced PDT have been conducted to date and have demonstrated the safety and feasibility of this treatment modification 12, 67. Although these latter clinical investigations were only designed to assess safety, enhancements in tumor clearance were observed both clinically and histologically when CP94 was included within the photosensitising cream.

A more recent development in this field has been the design and synthesis of a new iron chelating PpIX prodrug, AP2‐18 (Patent: Curnow, Wood & Perry; 06/03/14; PCT/GB2013/052297) 70. This experimental drug essentially ester links ALA to CP94 to aid their co‐delivery to cells in equimolar amounts. This experimental study therefore aims to compare the effect of this new combined iron chelating PpIX prodrug in the MRC5 and A431 cell types previously studied with CP94 with success 53. The levels of PpIX fluorescence produced by AP2‐18 as well as the PDT effects (as assessed by the lactate dehydrogenase and neutral red assays) produced within each cell type following subsequent irradiation have therefore been compared against the established PpIX precursors ALA and MAL administered either alone, or in combination with, the most effective known iron chelating agent for this purpose, CP94.

## MATERIALS AND METHODS

All media, reagents, and chemicals were obtained from Sigma–Aldrich Chemicals Company (Poole, UK) unless otherwise stated.

### Cell Culture

Human fetal lung fibroblasts (MRC‐5) and human epithelial squamous carcinoma cells (A431) were purchased from the European Collection of Cell Cultures (ECACC, Wiltshire, UK). MRC‐5 and A431 cells were cultured in Eagle's minimum essential medium (EMEM) supplemented with 10% fetal bovine serum (FBS), 2% (200 mM) L‐glutamine and 2% (200μ ml^−1^) penicillin and (200 μg ml^−1^) streptomycin solution. All cell culture procedures were conducted in a class II laminar flow cabinet using aseptic conditions. The cells were grown in an incubator at 37°C and 5% CO_2_ and passaged as required. These two cell types were specifically selected for this investigation as they were the only cell types from a range that we have previously studied 53, where we observed iron chelation significantly enhancing the effects of PpIX‐PDT.

### Test Compound Preparation

A total of 100 mM stock solutions of each test compound (ALA, MAL, CP94 [Professor Hider, King's College London, UK] and AP2‐18 [Drs Wood & Perry, University of Exeter, UK]) were prepared by dissolving the individual powders in PBS. These were stored at 5°C for up to one month. Further dilution to the final concentrations employed (500 and 1,000 μM) for each compound was achieved using colorless modified EMEM (i.e., without phenol red; Fisher Scientific, UK) on the day of each experiment. These specific concentrations were selected as previous experimentation 53 has indicated that similar quantities of PpIX are produced by 500 μM ALA and 1,000 μM in these particular cell types when incubated over the time period of interest (3–6 hours).

### Cell Preparation and Test Compound Incubation

A cell count was performed utilising trypan blue before seeding the cells into 96‐well plates (Corning, flat clear‐bottom, black [to prevent fluorescence bleeding into neighboring wells]) at a density of 4.3 × 10^4^ cells per well. Cell viability as determined by the trypan blue exclusion method was found to be >98% for all experiments. The cells were then incubated overnight (37°C; 5% CO_2_) and had reached confluence the next day, ready for experimentation. The medium was then removed, the adherent cells washed three times with phosphate‐buffered saline (PBS) and 100 μl of the test solutions (ALA ± CP94, MAL ± CP94 or AP2‐18) introduced as required to the individual wells. This part of the procedure, as well as the rest of the experiment, was conducted under reduced ambient light levels within the laboratory to reduce the occurrence of PpIX photobleaching. In each instance, two separate plates were set up exactly in parallel—one for irradiation and the other to act as a dark control.

### PpIX Fluorescence Quantification

PpIX fluorescence was measured using the SkanIt software on the VarioskanFlash plate reader (Thermo Fisher Scientific, Finland). Measurements were taken every hour for 6 hours to cover the drug‐light period usually adopted within clinical practice, with 405 ± 12 nm excitation and 635 ± 12 nm emission filters in place to measure the PpIX fluorescence produced. This experimentation was proceeded by the production of a standard curve utilizing synthetic PpIX (data not shown; see also Ref. 53). Between measurements, plates were kept in the incubator (37°C; 5% CO_2_) with care being taken throughout to avoid any PpIX photobleaching.

### Light Irradiation

Immediately following the PpIX fluorescence measurement after 6 hours, the treatment solutions were removed from all the wells and replaced with 150 μl modified EMEM plus 5% FBS. This step ensured that the subsequent cell death observed following irradiation was solely due to the PpIX accumulated inside the cells themselves. The irradiation plate was then placed 5 cm below an Aktilite CL16 LED array (Galderma, UK) and irradiated with 30 J/cm^2^ of red light (635 ± 2 nm; 75 mW/cm^2^). The other parallel plate was kept in the dark, out of the incubator, for the same amount of time to act as a dark control. The duration of this irradiation period, with this particular light source, was just under 8 minutes. Following irradiation, a final PpIX fluorescence reading was undertaken prior to incubating the cells overnight (37°C; 5% CO_2_).

### Quantification of PDT Effectiveness

The cytoTox 96® assay (Promega, UK) was utilized to determine the PDT effect of each set of parameters on each cell type. This assay colorimetrically measures the amount of lactate dehydrogenase (LDH) released by lysed cells into the medium, by supplying lactate, NAD^+^ and a tetrazolium salt (iodonitrotetrazolium violet; INT) as substrates in the presence of the diaphorase. The amount of the red formazan product generated by this enzymatic reaction was proportional to the amount of LDH released and therefore the number of dead cells 71. Specifically, 12 hours following irradiation the plates were centrifuged (200 *g* for 5 minutes) and 50 μl of each well's supernatant transferred to a clean plate. A 50 μl cytoTox 96® reagent was added to each new well and the whole plate was kept in the dark at room temperature for 30 minutes before the reaction was terminated. A plate reader (VarioskanFlash) was employed to read absorbance at 490 nm. In addition to the test compounds, each plate also contained three wells with completely lysed cells to act as a (positive) maximum LDH release control, so that the percentage cell death could be derived.

These LDH observations of PDT effectiveness were also confirmed using the neutral red assay, which measures the ability of intact cells to take up neutral red *via* active transport mechanisms and incorporate it within their lysosomes. Subsequently, following washing, the viable cells in each sample can release this bound dye (under acidified‐extracted conditions) for quantification to determine the total number of viable cells present 72. Specifically, 50 μl colorless EMEM plus 7.5 μl neutral red solution was added to each well of the original plates following their centrifugation and supernatant removal above and incubated for 1 hour (37°C; 5% CO_2_). The wells were then washed once with 150 μl PBS and 150 μl solubilization solution added per well and left for 10 mins before pipetting up and down gently (to release all neutral red). Each plate also included three wells with completely lysed cells to act as a positive control. A plate reader (VarioskanFlash) was then employed to read the absorbance generated at 540 nm to determine the number of cells that survived the PDT treatment.

### Statistical Analysis

Each experiment was conducted in triplicate (three separate wells) on three different days. Each plate also contained (blank) control cells incubated without the test compounds, which were utilized to remove background fluorescence from the readings of the test compounds detailed above. Results were presented as mean normalized PpIX fluorescence, percentage cell death (LDH assay) and percentage cell survival (neutral red assay) ±standard error (SE). Statistical significance (indicated at the *P* < 0.05 level) was determined using an independent samples analysis of variance (ANOVA) to compare the differences in PpIX fluorescence produced by the different test compounds at each time point. A repeated measures ANOVA was also conducted to compare the overall difference in PpIX fluorescence produced by ALA and MAL.

## RESULTS

### Baseline Comparison of ALA Alone Versus MAL Alone

**Figure 1:**
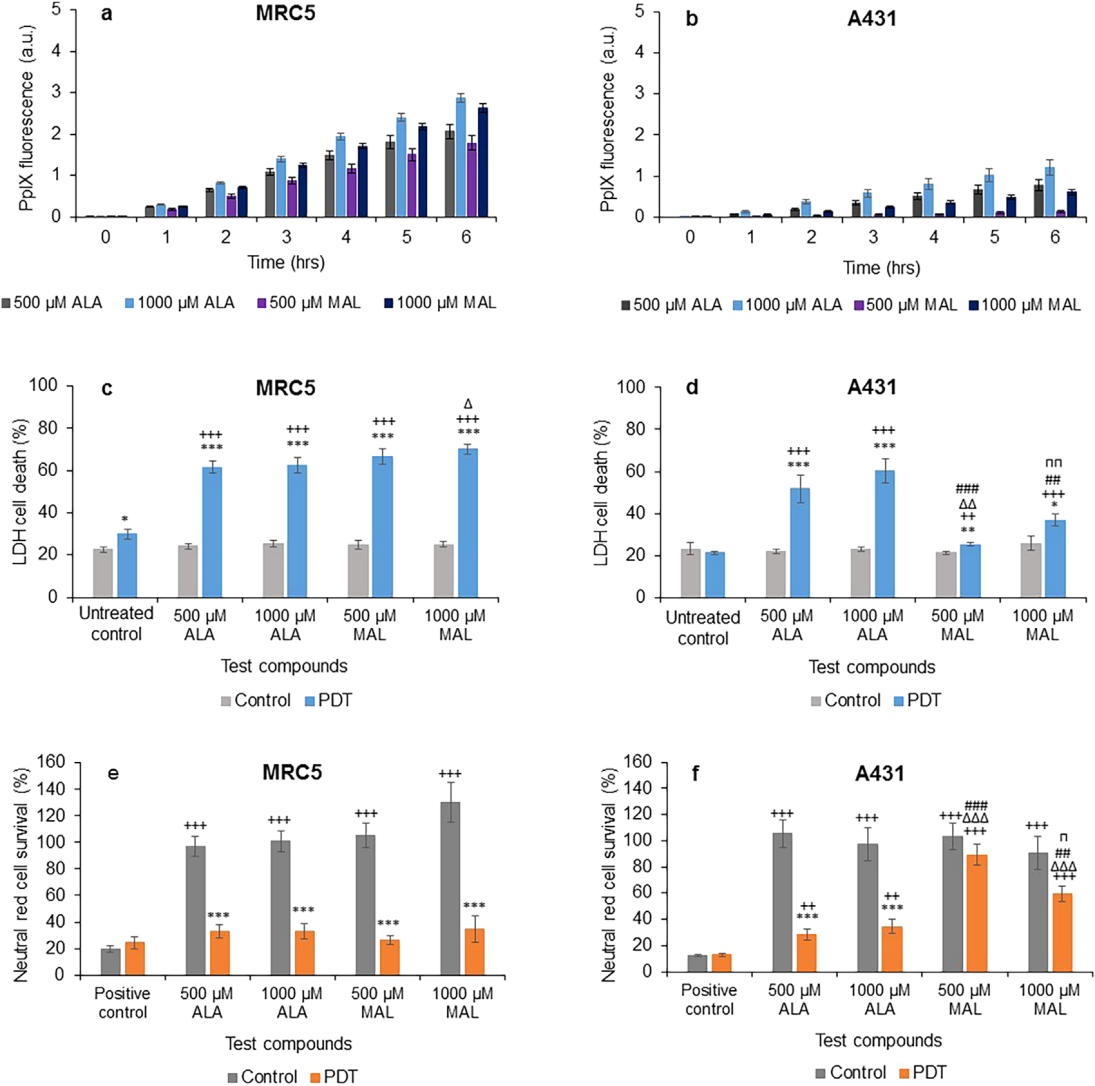
Mean (±SE) PpIX fluorescence in (**a**) lung fibroblasts (MRC5) and (**b**) squamous cell carcinoma (A431), treated with 500 or 1,000 μM of ALA or MAL for 6 hours. Results also show corresponding percentages of LDH cell death of MRC5 (**c**) and A431 (**d**), and Neutral red cell survival of MRC5 (**e**) and A431 (**f**) respectively (±SE), of irradiated cells (PDT) compared to the non‐irradiated (control) cells. Statistically significance (ANOVA) to no light control = *, to positive/untreated control = +, to 500 μM ALA = Δ, to 1,000 μM ALA = #, and to 500 μM MAL = *n*. * = *P* < 0.05, ** = *P* <0.01, and *** = *P* < 0.001.

PpIX fluorescence was measured hourly for 6 hours after treating the cells with 500 or 1,000 μM ALA or MAL. PpIX fluorescence was observed to increase over time as expected for both cell types investigated. The normal lung fibroblasts (MRC5) were noted to accumulate more PpIX in arbitrary units compared to the squamous cell carcinoma cells (A431) (Figs. 1a and b). For both cell types investigated here, 1,000 μM ALA consistently produced the highest level of fluorescence observed, followed by 1,000 μM MAL and then 500 μM ALA in the normal fibroblasts (Fig. 1a) but 500 μM ALA and then 1,000 μM MAL in squamous carcinoma cells (Fig. 1b), indicating that ALA was a more effective PpIX prodrug than MAL in this cell type. Overall, the PpIX fluorescence produced by each congener were significantly different (*P* < 0.05) to one another in both cell types.

As anticipated, the percentage of cell death in the irradiated (PDT) groups was observed to be significantly higher than the corresponding non‐irradiated (dark) controls with both cell types (Figs. 1c and d). In the irradiated PDT group of both cell types, addition of the PpIX prodrugs ALA or MAL led to a significantly increased cell death compared to the corresponding negative controls. In the fibroblast PDT group, only 1,000 μM MAL produced significantly higher cell death compared to 500 μM ALA (Fig. 1c). In the carcinoma PDT group however, 500 μM MAL produced significantly less cell death compared to 500 μM ALA or 1,000 μM ALA, while 1,000 μM MAL produced significantly less cell death than 1,000 μM ALA but significantly more than 500 μM MAL (Fig. 1d). These measures of cell death obtained with the LDH assay were therefore in close alignment with the PpIX fluorescence measurements observed above (i.e., bigger PDT effects generally observed in the groups determined to have the highest PpIX levels prior to irradiation).

PDT effectiveness was further confirmed using the neutral red assay, which determined cell survival. The results supported that recorded with the LDH assay above, with significantly fewer cells being able to incorporate neutral red in their lysosomes being observed in the PDT group than the (dark) control group in all cases (Figs. 1e and f). In the squamous carcinoma PDT groups, ALA was also observed to be a more effective photosensitising agent in this cell type than MAL. Administration of 500 μM MAL was associated with the highest percentage of cell survival observed after irradiation (i.e., lowest cell kill) when compared to the other test compounds (Fig. 1f) and so was the least effective variable investigated in this particular series of experimentation.

### Comparison of ALA Alone, ALA ± CP94, and AP2‐18

**Figure 2:**
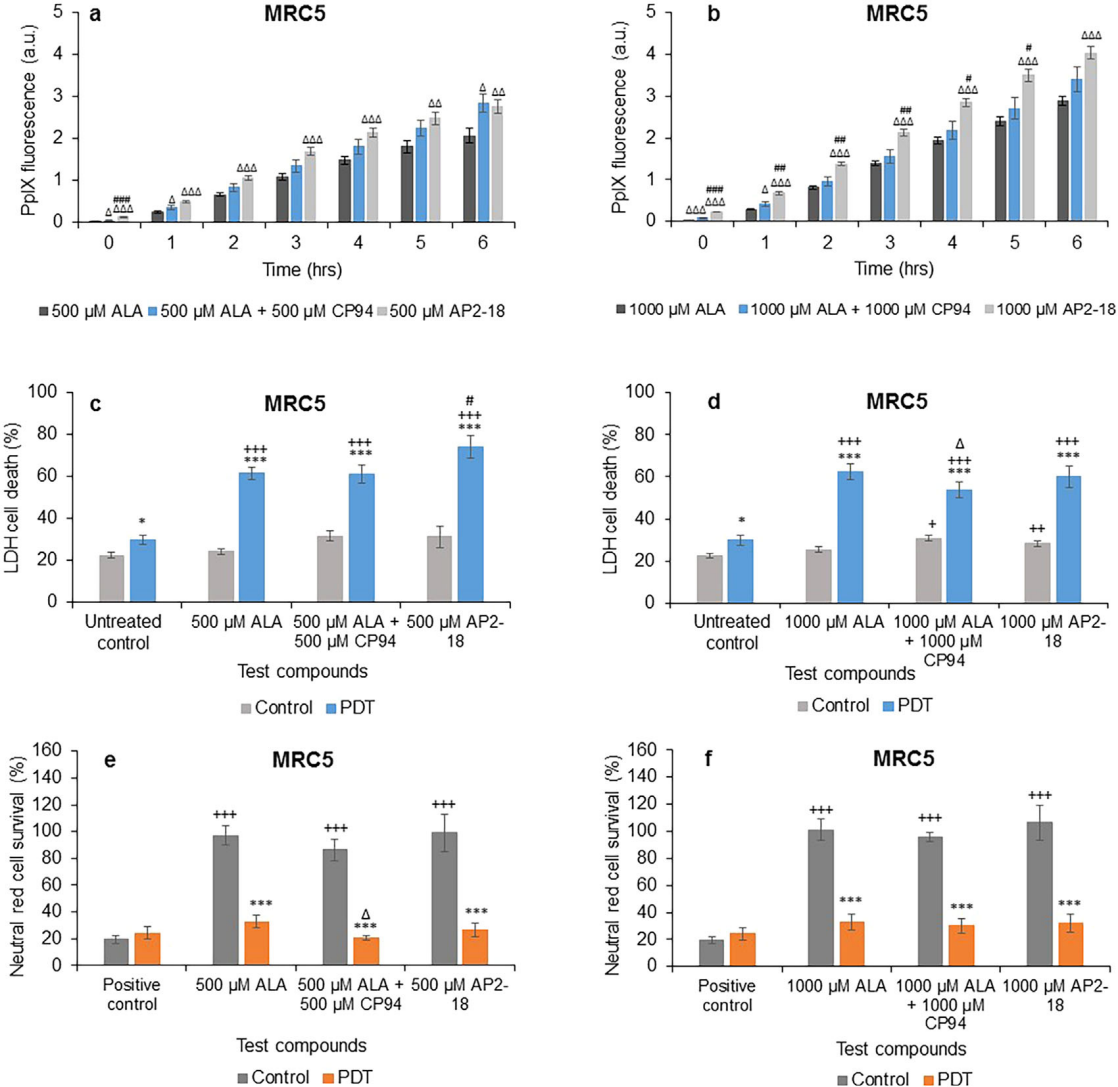
Mean (±SE) PpIX fluorescence in lung fibroblasts (MRC5) treated with (**a**) 500 μM or (**b**) 1,000 μM of ALA ± CP94 or AP2‐18 for 6 hours. Results also show corresponding percentages of LDH cell death (**c**) and (**d**), and Neutral red cell survival (**e**) and (**f**) respectively (±SE), of irradiated cells (PDT) compared to the non‐irradiated (control) cells. Statistically significance (ANOVA) to no light control = *, to positive/untreated control = +, to MAL = Δ, and to MAL ± CP94 = #. * = *P* < 0.05, ** = *P* < 0.01, and *** = *P* < 0.001.

In normal fibroblasts, addition of CP94 to ALA at both doses investigated was observed to increase PpIX fluorescence slightly but only with statistical significance at 1 hour, and then also at 6 hours for the 500 μM group (Figs. 2a and b). Using the new combined iron chelating PpIX prodrug AP2‐18 however, significantly increased PpIX fluorescence compared to ALA alone for all six time points at both dose investigated (Figs. 2a and b). Furthermore, it was noted that utilizing 1,000 μM AP2‐18 also produced significantly more PpIX fluorescence than 1,000 μM ALA and 1,000 μM CP94 administered separately as a combination for the first 5 hours (Fig. 2b). PpIX accumulation, as monitored *via* fluorescence, increased as expected over this time period in all experimental groups.

Cell death of irradiated normal fibroblasts was observed with the LDH assay to be significantly higher than that of the non‐irradiated cells, and to the negative control for both concentrations (Figs. 2c and d). In the 500 μM PDT group, AP2‐18 produced significantly more cell death compared to ALA + CP94 administered separately in combination (Fig. 2c). In the 1,000 μM dosed groups, ALA + CP94 produced significantly less cell death compared to ALA alone in irradiated cells, while ALA + CP94 and AP2‐18 produced significantly more cell death in the non‐irradiated cells (Fig. 2d). These findings mirror our previous work where enhancement of PpIX‐induced PDT with an iron chelating agent was observed to be more effective at lower (more limited) doses of the PpIX precursor 73. The neutral red assay results, although less discerning between the various parameters investigated, were supportive of the LDH assay findings and consistently confirmed significantly less cell survival in the PDT groups than the control groups (Figs 2e and f), with 500 μM ALA + 500 μM CP94 producing significantly more PDT effectiveness compared to 500 μM ALA alone (Fig. 2e).

**Figure 3:**
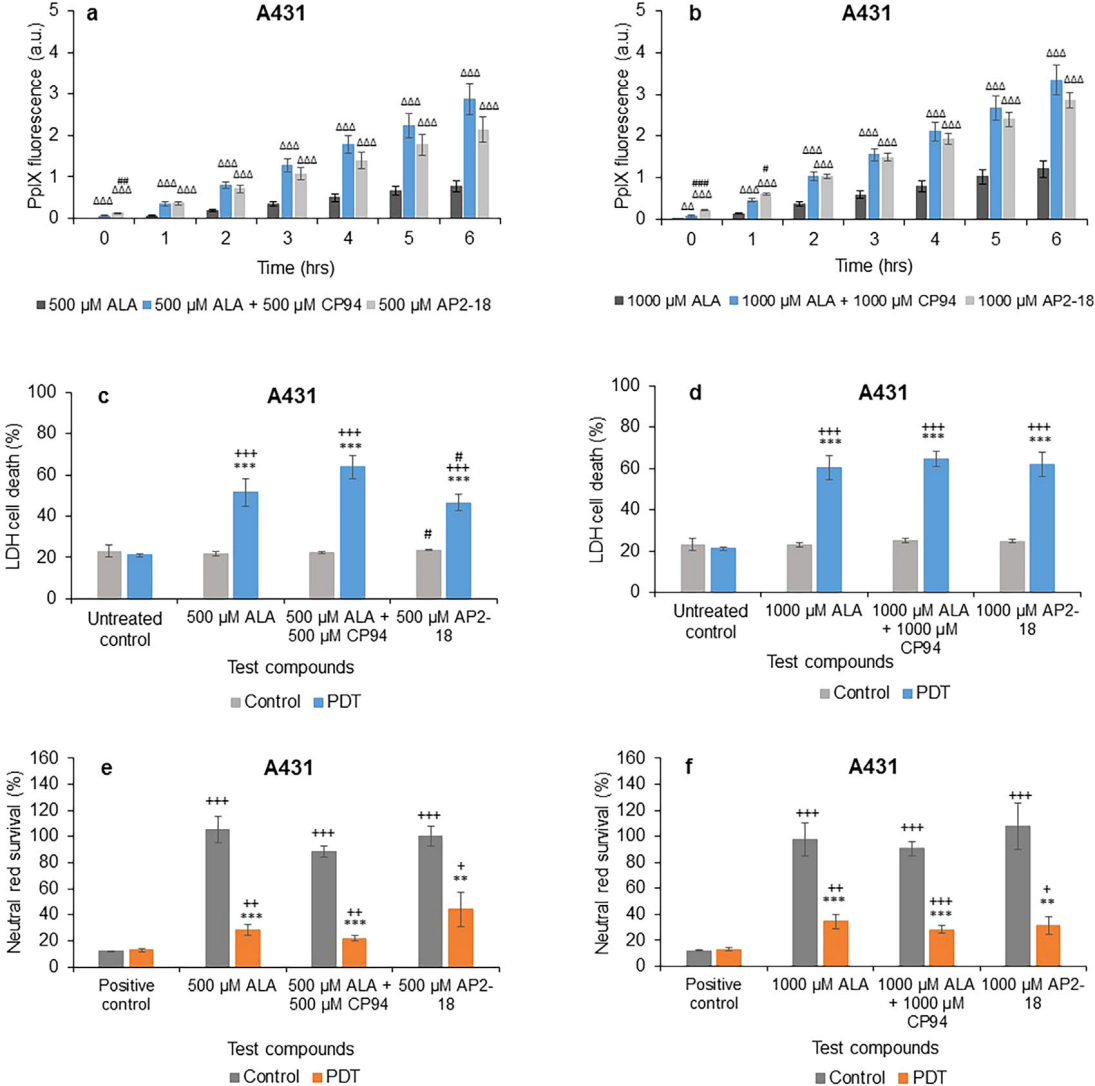
Mean (±SE) PpIX fluorescence in squamous cell carcinoma cells (A431) treated with (**a**) 500 μM or (**b**) 1,000 μM of ALA ± CP94 or AP2‐18 for 6 hours. Results also show corresponding percentages of LDH cell death (**c**) and (**d**), and Neutral red cell survival (**e**) and (**f**) respectively (±SE), of irradiated cells (PDT) compared to the non‐irradiated (control) cells. Statistically significance (ANOVA) to no light control = *, to positive/untreated control = +, to ALA = Δ, and to ALA ± CP94 = #. * = *P* < 0.05, ** = *P* < 0.01, and *** = *P* < 0.001.

In squamous carcinoma cells, PpIX fluorescence also increased over time as expected. Addition of CP94 to the ALA prodrug administration or alternatively utilising AP2‐18, significantly increased PpIX fluorescence compared to ALA alone at all time points for all concentrations (Figs. 3a and b). Thus demonstrating the beneficial effect of enhanced PpIX accumulation *via* the approach of using an iron chelating agent to augment this treatment modality. Although PpIX fluorescence in ALA + CP94 treated cells with both doses investigated was observed to be slightly higher than that in AP2‐18 treated cells (Figs. 3a and b), these differences were not found to be significant on statistical analysis.

Cell death as determined by the LDH assay was once again observed to be significantly higher in the PDT group compared to the control group as well as in the treated cells compared to the negative control in the PDT group for all concentrations with the squamous carcinoma cells (Fig. 3c and d). AP2‐18 produced significantly less cell death in this cell type (A431) compared to ALA + CP94 administered separately in combination in the 500 μM PDT group, but significantly more cell death compared to ALA + CP94 in the control group (Fig. 3c) indicating a minor increase in dark toxicity with these particular experimental parameters. When utilizing the higher dose of 1,000 μM, no significant difference was observed between the cytotoxic effects of the different PDT groups as ascertained with the LDH assay. The neutral red results indicated significantly lower cell survival (and therefore increased cell kill) in irradiated cells compared to non‐irradiated cells (Figs. 3e and f) with no significant differences being observed between the different PDT groups in this cell type.

### Comparison of MAL Alone, MAL ± CP94, and AP2‐18

**Figure 4:**
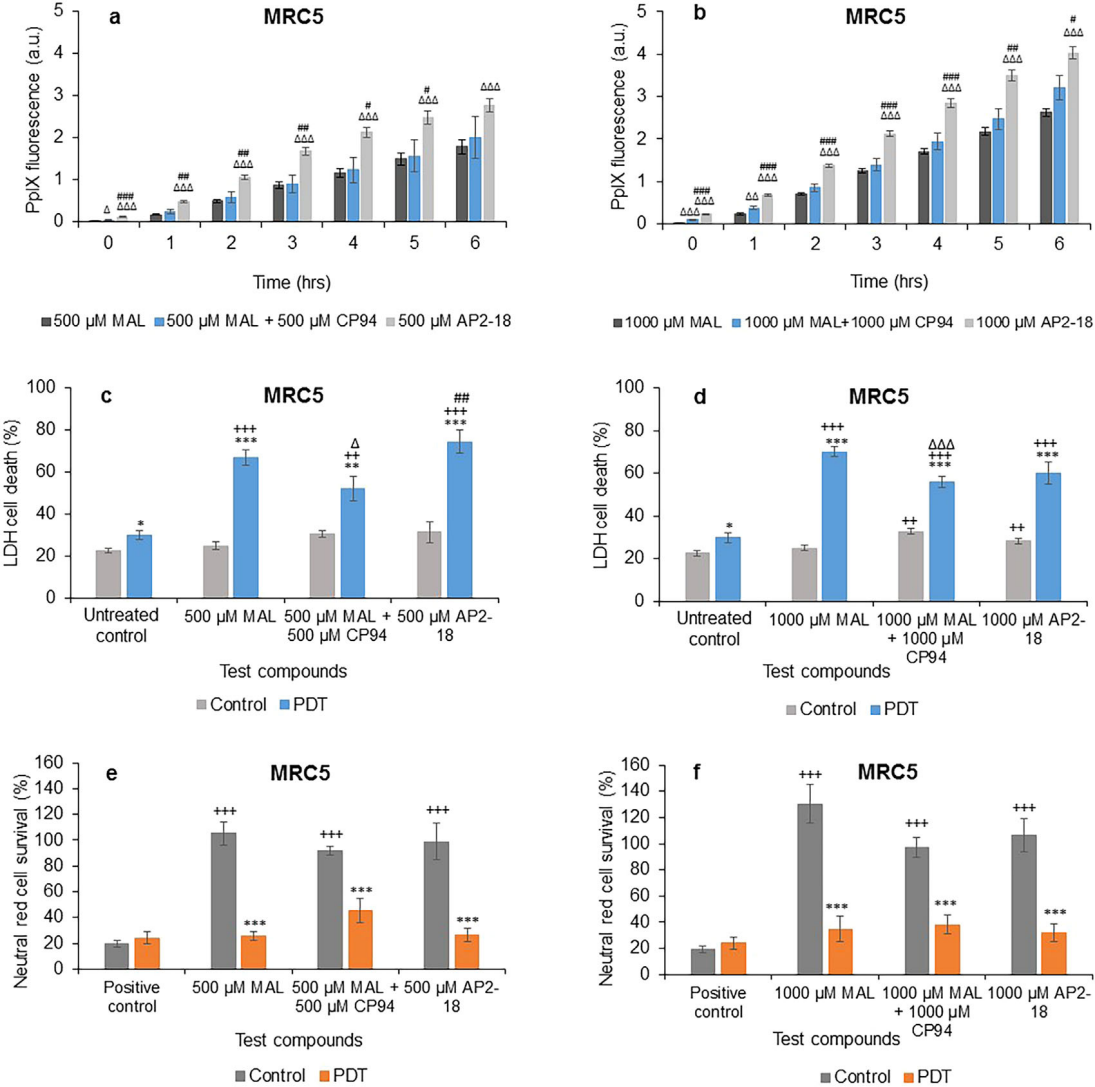
Mean (±SE) PpIX fluorescence in lung fibroblasts (MRC5) treated with (**a**) 500 μM or (**b**) 1,000 μM of MAL ± CP94 or AP2‐18 for 6 hours. Results also show corresponding percentages of LDH cell death (**c**) and (**d**), and Neutral red cell survival (**e**) and (**f**) respectively (±SE), of irradiated cells (PDT) compared to the non‐irradiated (control) cells. Statistically significance (ANOVA) to no light control = *, to positive/untreated control = +, to MAL = Δ, and to MAL ± CP94 = #. * = *P* < 0.05, ** = *P* < 0.01, and *** = *P* < 0.001.

In normal fibroblasts, addition of CP94 to MAL did not statistically increase the normal pattern of increasing PpIX fluorescence over 1–6 hours for both concentrations, except for at 1 hour in the 1,000 μM group (Figs. 4a and b). Using AP2‐18 in this cell type however, significantly increased PpIX fluorescence compared to MAL alone at all time points for both concentrations, as well as when compared to MAL + CP94 at all time points considered with the 1,000 μM dose parameter (Fig. 4b) and all time points up to and including 5 hours with 500 μM administration (Fig. 4a). Thus indicating the enhanced effect of this new iron chelating PpIX prodrug on PpIX accumulation over and above that of MAL in this cell type.

Normal fibroblast cell death determined *via* the LDH assay in irradiated cells was again significantly higher than within the non‐irradiated groups and the negative control for all concentrations employed (Figs. 4c and d). In the 500 μM group, AP2‐18 produced significantly more cell death when compared to MAL + CP94, but not to MAL alone (Fig. 4c) and the MAL + CP94 group produced significantly less cell death than the MAL alone group on this occasion (Fig. 4c). In the 1,000 μM dosed PDT groups, MAL + CP94 also produced significantly less cell death compared to MAL alone, with MAL alone and AP2‐18 not producing statistically different results from one another. In the control group, both MAL + CP94 and AP2‐18 produced significantly more cell death compared to the negative control (Fig. 4d). The corresponding neutral red results (Figs. 4e and f) verified that the PDT initiated on irradiation significantly decreased cell survival compared to the non‐irradiated cells, and agreed with the LDH assay results. No significant differences were observed between the cell kill achieved with MAL alone, MAL + CP94 or AP2‐18 in this cell type.

**Figure 5:**
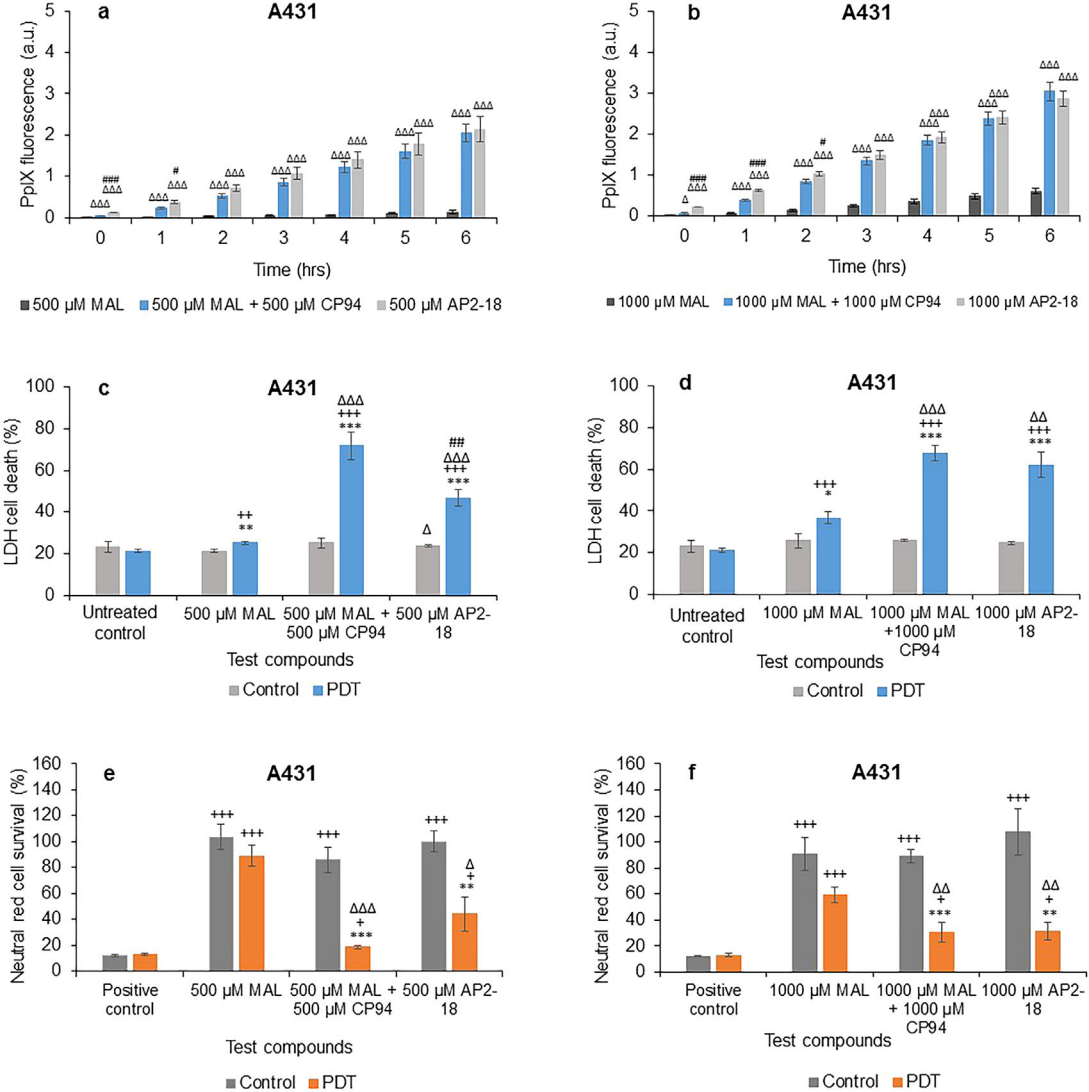
Mean (±SE) PpIX fluorescence in squamous cell carcinoma cells (A431) treated with (**a**) 500 μM or (**b**) 1,000 μM of MAL ± CP94 or AP2‐18 for 6 hours. Results also show corresponding percentages of LDH cell death (**c**) and (**d**), and Neutral red cell survival (**e**) and (**f**) respectively (±SE), of irradiated cells (PDT) compared to the non‐irradiated (control) cells. Statistically significance (ANOVA) to no light control = *, to positive/untreated control = +, to MAL = Δ, and to MAL ± CP94 = #. * = *P* < 0.05, ** = *P* < 0.01, and *** = *P* < 0.001.

In squamous carcinoma cells, addition of CP94 to MAL or using AP2‐18 once again significantly increased the increasing PpIX fluorescence observed at all time points investigated compared to administration of MAL alone (Figs. 5a and b). PpIX fluorescence in AP2‐18 treated cells compared to MAL + CP94 treated cells was observed to be significantly higher up to 1 hour in the 500 μM group (Fig. 5a) and up to 2 hours in the 1,000 μM group (Fig. 5b) indicating that the rate of accumulation may have been slightly faster at the beginning of the drug‐light interval with the new iron chelating PpIX prodrug, AP2‐18.

Cell death in irradiated squamous carcinoma cells using the LDH assay was found to be significantly higher compared to the non‐irradiated cells, as well as to the negative control in the PDT group for all concentrations employed (Figs. 5c and d). However, both MAL + CP94 and AP2‐18 produced significantly more cell death compared to MAL alone in the PDT groups for all concentrations (Figs. 5c and d), indicating that the approach of iron chelation was particularly advantageous when considering undertaking MAL‐PDT in this skin cancer cell type. AP2‐18 produced significantly less cell death compared to MAL + CP94 in the 500 μM PDT group and significantly more cell death than MAL alone in the control group (Fig. 5c). The neutral red results verified the LDH findings with MAL + CP94 and AP2‐18, indicating the production of significantly lower cell survival (and thus more cell kill) compared to MAL alone for all concentrations (Figs. 5e and f). Furthermore, these experimental groups (MAL + CP94 vs. AP2‐18) were not found to produce any significantly different effects to one another, despite their different compositions.

## DISCUSSION

PDT using topical prodrugs requires the conversion of these PpIX precursors into the active photosensitizer 74. The highest dose of ALA investigated here (1,000 μM) consistently produced the highest level of PpIX fluorescence observed, indicating that ALA was a more effective PpIX prodrug than MAL in both the cell types investigated (Figs. 1a and b). This supports our previous findings where MAL‐treated cells accumulated less PpIX than cells treated with the same concentration of ALA 55. Furthermore the cytotoxicity findings of cell death quantified with the LDH assay (Figs. 1c and d) were in close alignment with the PpIX fluorescence measurements (i.e., the greatest cell death was generally observed in the groups determined to have the highest PpIX levels prior to irradiation). This agrees with the published findings of others, which have clearly demonstrated that increased PpIX accumulation leads to increased cell death post irradiation 56 and that the degree of cell damage produced depends on the amount of PpIX accumulated pre‐irradiation 30. PDT effectiveness was further confirmed using the neutral red assay (Figs. 1e and f) and mirrored the results observed with the LDH assay.

In the squamous carcinoma cells, ALA was again observed to be a more effective photosensitizing agent than MAL in this experimental system, although generally it was noted that in this particular series of experimentation that the normal fibroblasts accumulated more PpIX, which resulted in bigger PDT cell kills on irradiation, than with the squamous carcinoma cells. This contradicts some studies which suggest that cancerous cells make more PpIX than normal cells 56, 75 because of their higher metabolic activity 54 and their altered iron metabolism 12. PpIX is thought to accumulate to a greater degree and for a longer period of time in abnormal cells than normal cells 9. De Souza et al. however, found that different cell lines produced different amounts of PpIX, most likely due to their differences in metabolism 54. They found no correlation between cell proliferation and production of PpIX 54. They suggest that PpIX production depends on the amount of enzymes present and this is associated with the number of mitochondria in each cell 54. This could help explain the difference in the levels of PpIX seen within the different cell types investigated here, as the MRC5 cells are larger than the A431 cells and would therefore contain many more mitochondria and hence more enzymes.

These observations also reinforce the importance of including more than one cell type in experimental investigations of this nature, despite the compromise of having to include the normal lung fibroblasts alongside the more dermatological pertinent squamous cell carcinoma cells of interest. However, the other non‐cancerous dermatological cell types we have investigated in the past 53, including normal skin fibroblasts (84BR) and keratinocytes (NHEK) only demonstrated minimal enhancement when investigating the iron chelating agents DFO and CP94 to augment ALA‐ or MAL‐induced PDT. Nonetheless, these findings will be beneficial if they translate into clinical practice, where avoidance of excessive damage to healthy cells in the treatment site is a desirable outcome.

Adding equimolar concentrations of the iron chelator CP94 to the PpIX precursors increased PpIX accumulation in all cell types, but only significantly in the cancer cells. This suggests that the iron chelator CP94 is most effective in the cancer cells where the haem biosynthesis pathway is more likely to be disrupted 7, 76. These findings support several studies that found that the addition of an iron chelator to a PpIX precursor increased PpIX accumulation and therefore fluorescence before irradiation 26, 55, 56, with MAL + CP94 treated cells producing the greatest enhancement of PpIX fluorescence in tumor cells 12, 20. This occurs because iron chelating agents reduce cellular levels of free iron, which is essential for haem formation to take place and thus this change in cellular environment, forces more PpIX to accumulate in the cell 54, 56, 76.

Although MAL is more lipophilic 6, 26 and has a shorter time of photosensitivity 34 compared to ALA, which may convey an advantage in clinical application to skin lesions, after MAL absorption has occurred, the additional methyl group needs to be removed *via* cytosolic esterases before it can enzymatically converted into PpIX. This adds an additional step to PpIX production from this PpIX precursor, which may reduce the rate of its PpIX accumulation slightly in experimental cellular systems as it will depend on specific cell types’ esterase capabilities. Furthermore, it is known this small difference (the addition of a methyl group) also affects the route of drug entry into cells. ALA is known to enter cells *via* active transport mechanisms *via* GABA transporters, whereas MAL is thought to enter cells *via* passive diffusion and non‐polar amino acid transport 77.

In the normal fibroblasts, using the new combined iron chelating PpIX prodrug AP2‐18 not only significantly increased the PpIX fluorescence produced compared to ALA alone for all six time points at both doses (Figs. 2a and b), the highest dose explored (1,000 μM AP2‐18) also produced significantly more PpIX fluorescence than 1,000 μM ALA and 1,000 μM CP94 administered separately. This latter finding was unexpected and it is hypothesized that this was most likely due to the co‐delivery of the two component parts of the drug being delivered to the cell simultaneously before the action of cytosolic esterases separated them to affect PpIX accumulation more effectively, than possible with the separately administered counterparts. On irradiation however, only the increased PpIX levels produced by 500 μM AP2‐18 translated into greater PDT effects than 500 μM ALA and 500 μM CP94 administered separately (Fig. 2c). This particular finding supports our previous work where enhancement of PpIX‐induced PDT with an iron chelating agent has been observed to be more effective at lower (and thus more limited) doses of the PpIX precursor, rather than at higher levels where PpIX may already be present in abundance 73.

Although it is very difficult to translate experimental findings with adherent monolayers of a single cell type into the real world challenge of reaching the bottom of thicker nBCC tumors in clinic, it is clear from our fluorescence microscopy findings of this clinical situation 18 that PpIX levels at the base of these tumors can indeed be inadequate. We therefore postulate that although there is light reaching this depth and there is molecular oxygen present, there is currently insufficient PpIX to cause cataclysmic oxidative stress and thus cell death in this situation with the current licensed dermatological PDT treatment regime 10. However, as observed with CP94 in our clinical pilot studies both with ALA and MAL [67 and 12, respectively], the addition of an iron chelating agent can actually produce complete clinical clearance of nBCC with a single treatment cycle. This is due to more PpIX being able to accumulate throughout the thickness of the tumor (NB: not more PpIX production, just greater accumulation of that PpIX produced from the same amount of precursor substrate), whilst the presence of an effective iron chelator temporarily prevents the conversion of PpIX to haem. This approach (iron chelation) essentially makes the PpIX already being produced at the lower levels of thicker tumors have more utility at the time of irradiation. The finding that the new combinational iron chelating PpIX prodrug AP2‐18 may also be able to operate in a similar manner in this respect to CP94, particularly when PpIX levels are more limited is therefore encouraging and warrants further detailed study. This is particularly important as CP94 is already in the public domain, which means it cannot be protected in a secure manner for clinical development. The new combined PpIX‐precursor and siderophore, AP2‐18 has therefore been specifically developed for this application and its synthesis patented 70 to resolve this issue, so that the enhancement of PpIX‐induced PDT *via* iron chelation can be developed clinically for patient benefit.

Incubation of normal fibroblasts with AP2‐18 was also found to significantly increase the PpIX fluorescence levels observed, when compared to MAL alone at all time points for both concentrations investigated, as well as compared to MAL + CP94 at all time points up to and including 5 hours with both doses considered (Fig. 4). This indicated the enhanced effect of this new form of iron chelating PpIX prodrug on PpIX accumulation over and above the action of MAL as a PpIX‐precursor for PDT in this cell type. However, no significant differences were observed between the PDT effect achieved with MAL alone, MAL + CP94 or AP2‐18 in this cell type with the neutral red and LDH assays recording a mixed picture with 500 μM AP2‐18 out‐performing MAL + CP94 but not MAL alone and the MAL + CP94 groups at both concentrations producing significantly less PDT effects than the corresponding MAL alone groups.

When this experimentation was repeated with the squamous carcinoma cells (Fig. 3) both the addition of CP94 to ALA or AP2‐18 administration significantly increased PpIX fluorescence compared to ALA alone at all time points for all concentrations in a similar manner but this increased accumulation failed to translate into greater PDT effectiveness over and above the already effective action of ALA alone in this cell type. However when MAL was utilized as the PpIX precursor (Fig. 5), iron chelation using CP94 addition to MAL or alternatively AP2‐18 administration not only significantly increased the increasing PpIX fluorescence observed at all time points investigated compared to administration of MAL alone but also produced significantly more cell death compared to MAL alone for both concentrations in a similar manner. This indicates that the approach of supplementing with a pyridinone iron chelator was particularly advantageous when undertaking MAL‐PDT in this skin cancer cell type. CP94 is known to enter cells *via* simple diffusion 78 and this property of AP2‐18 is yet to be determined but is likely to be similar to that of CP94. The results of this experimentation also provide an indication that ubiquitous cytosolic esterases also manage to effectively separate the ALA and CP94 components that comprise AP2‐18 successfully within the cell types employed here (in a similar manner to the removal of the methyl group from MAL), so that the normal but elevated biochemical processes can ensue.

PpIX is produced in the mitochondria, but it can subsequently localize within cell membranes and the cytosol 5, 79, leading to apoptosis or necrosis respectively 8. We previously found that cell death following PDT resulted from low amounts of apoptosis but high amounts of necrosis, possibly as a result of huge mitochondrial damage 20. However, the main type of cell death following PDT is still to be established and heavily depends on the treatment parameters employed 20. For this study, the lactate dehydrogenase assay was used to measure cell death. This assay measures LDH release, which occurs following the rupture of the cell membrane. This process usually occurs during necrosis. However, the cells may have also undergone some form of programmed cell death, which can occur without membrane rupture and hence LDH detection *via* this method. This aspect should therefore be investigated in more detail in future experimentation, despite validating the LDH assay findings with concurrent assessment of the number of living cells able to actively take up neutral red. It is also important to note that AP2‐18 as well as ALA, MAL, and CP94 were all found to produce minimal dark toxicity throughout this study.

In summary, generally iron chelation achieved *via* CP94 or AP2‐18 administration significantly increased PpIX fluorescence. ALA was again found to be a more effective PpIX‐prodrug than MAL in A431 cells 55, corresponding with the lower PpIX accumulation observed with the latter congener. Addition of either iron chelator consistently increased PpIX accumulation but did not always convey an extra beneficial effect on PpIX‐PDT cell kill in both cell types when using the already highly effective ALA treatment parameters and the highest doses considered but was highly beneficial in the skin cancer cells when compared with MAL administration alone. AP2‐18 was also at least as effective as CP94 + ALA/MAL co‐administration throughout and significantly better than CP94 supplementation at increasing PpIX fluorescence in MRC5 cells as well as at lower doses where PpIX accumulation was observed to be more limited. PpIX accumulation as a measurement and indicator for successful PDT therefore appears to be highly dependent on the type of cells under investigation and as a result, future work should consider the effectiveness of this approach in other (non‐dermatological) tumor types, as the findings reported here may not be simply extrapolated to all other PpIX‐PDT indications without corroborating evidence first being sought. The new iron chelating PpIX prodrug AP2‐18 was found however in this particular experimental model, to be at least as effective as CP94 co‐administration with a PpIX‐precursor and significantly better than CP94 supplementation in certain circumstances. However, before AP2‐18 can be considered for future clinical study, confirmation of PpIX‐PDT enhancement within *in vivo* models will be required, along with an analysis of its pharmacokinetics and metabolism, so that an optimal treatment protocol can be derived and thus investigated for clinical efficacy.

## CONCLUSION

PpIX fluorescence levels, as well as PDT effectiveness on irradiation were found to be significantly increased by pyridinone iron chelation, either *via* the addition of CP94 to the administration of a PpIX precursor or alternatively *via* the newly synthesized combined iron chelating PpIX prodrug, AP2‐18. The effect of the latter compound appears to be at least equivalent to, if not better than, the separate administration of its constituent parts, particularly when employing MAL to destroy skin cancer cells. AP2‐18 therefore warrants further detailed analysis, as it may have the potential to improve dermatological PDT outcomes in specific clinical indications currently requiring enhancement.
